# The Effect of Aging on Quality of Life in Acromegaly Patients Under Treatment

**DOI:** 10.3389/fendo.2022.819330

**Published:** 2022-02-03

**Authors:** Naoki Yamamoto, Shin Urai, Hidenori Fukuoka, Masaaki Yamamoto, Kenichi Yoshida, Masaki Suzuki, Hiroki Shichi, Yasunori Fujita, Keitaro Kanie, Genzo Iguchi, Yutaka Takahashi, Wataru Ogawa

**Affiliations:** ^1^ Division of Diabetes and Endocrinology, Department of Internal Medicine, Kobe University Graduate School of Medicine, Kobe, Japan; ^2^ Division of Diabetes and Endocrinology, Kobe University Hospital, Kobe, Japan; ^3^ Medical Center for Student Health, Kobe University, Kobe, Japan; ^4^ Department of Biosignal Pathophysiology, Kobe University Graduate School of Medicine, Kobe, Japan; ^5^ Department of Diabetes and Endocrinology, Nara Medical University, Kashihara, Japan

**Keywords:** acromegaly, quality of life, arthropathy, older patients, AcroQoL

## Abstract

**Context:**

With the increasing number of older patients with acromegaly, it is important to understand the effects of aging on the quality of life (QoL) in acromegaly.

**Objective:**

To investigate the factors associated with the QoL of older acromegaly patients.

**Design:**

This was a single-center, retrospective, cross-sectional study conducted between 2014 and 2019.

**Methods:**

Among 90 acromegaly patients at Kobe University Hospital, 74 who had completed the QoL evaluation under treatment were enrolled (age = 62.0 [50.7–70.0], female 52%). SF-36 and the AcroQoL questionnaire were used to quantify QoL. The patients were divided into two groups: the young and middle-aged group, aged <65 years (51.0 [46.0–59.2], n =42), and the older group, aged ≥65 years (70.5 [69.0–73.0], n =32). The factors associated with the QoL scores were analyzed using univariate and multivariate regression analyses.

**Results:**

The scores for the physical component summary of SF-36 were negatively associated with age (*P <*0.01), while those for the mental or role/social component summary were positively associated (*P <*0.01, *P* =0.03, respectively). In contrast, AcroQoL scores were not associated with age. However, the different factors were associated with lower AcroQoL scores; arthropathy and higher BMI in the older group (*P <*0.01, and *P* =0.01, respectively), and treatment modalities and size of pituitary tumor in the young and middle-aged group (*P <*0.01, *P* =0.04, respectively). Replacement of hydrocortisone was commonly associated both in young and middle-aged group (*P* =0.04), and in older group (*P* =0.02).

**Conclusion:**

We showed that the factors associated with impaired QoL differed in the young and middle-aged, and older patients with acromegaly. In older patients, arthropathy and higher BMI were associated with poor QoL. These suggest the importance of early diagnosis and appropriate treatment in preventing arthropathy in acromegaly.

## Introduction

Acromegaly is a chronic condition characterized by the persistent excess of growth hormone (GH) and insulin-like growth factor-I (IGF-I) mainly caused by GH-secreting pituitary adenomas (1). This hormonal excess leads to multiple systemic complications, including metabolic disorders, cardiovascular disease, respiratory disease, osteoarthropathy, neoplasms, and a distinctive appearance. Moreover, the local mass effect around the sella turcica can cause several dysfunctions, such as visual impairment, ocular motility disorder, headache, and continuing hypopituitarism (2, 3). These complications lead to increased mortality, if not properly treated.

Advances in diagnostic approaches, surgical techniques, and pharmacological therapies for acromegaly have improved mortality in recent decades (4, 5). Therefore, the proportion of older patients with acromegaly is steadily increasing, as expected (6). However, the quality of life (QoL) of patients with acromegaly is still impaired even in a biochemically cured state (7). Improving QoL is considered the next step in managing this chronic disease and a major concern, especially for older patients. Older patients frequently have more complications, partly due to the lack of physiological reserves, which impair their daily living activity (8, 9) and lead to adverse treatment effects (10, 11). In a study of a healthy population using Short From-36 (SF-36), a widely used general health-related QoL questionnaire, QoL declined with age as a result of declines in physical, mental, and social functions (12). In a study using the AcroQoL questionnaire, a disease-specific questionnaire for acromegaly, some factors, including therapeutic modalities such as radiotherapy and drug therapy, symptoms such as headache and joint pain, and poor body image, contributed to poor QoL scores (13, 14). We have previously developed a Japanese version of AcroQoL and showed that acromegaly patients with surgical remission have better QoL scores than young and middle-aged patients receiving medical treatment or radiotherapy (15). However, the difference in QoL between young and middle-aged, and older patients remains unclear.

In the present study, to clarify the impact of aging on the QoL of patients with acromegaly under treatment, we compared the QoL scores in older patients with those in young and middle-aged patients and investigated the factors associated with QoL.

## Material and Methods

### Patients and Designs

This was a single-center, retrospective, cross-sectional study. Ninety patients with acromegaly who were visiting the Division of Diabetes and Endocrinology at Kobe University Hospital between March 2014 and September 2019 were included. Eighty-one patients completed the AcroQoL questionnaire. Among them, untreated patients (n =7) were excluded and, finally, 74 patients receiving treatment were enrolled in the present study. Among them, 61 patients completed the SF-36. The diagnosis of acromegaly was based on the diagnostic guidelines (16). Briefly, clinical signs, impaired serum GH suppression to less than 1.0 ng/mL during a 75 g oral glucose tolerance test, elevated serum IGF-I levels over the age- and sex-matched normal range, and the presence of pituitary tumors were confirmed in all the patients. The duration of the disease was defined as the period from the first diagnosis of the disease to the date the QoL questionnaire was administered. The clinical data of the patients were obtained from their medical records when the questionnaire was performed. The random GH (<1.0 ng/mL) and IGF-I standard deviation score (SDS) (<2.0) were used as the criteria for biochemical disease control as described previously (17). Only the IGF-I SDS, but not GH, was used in two patients treated with a growth hormone receptor antagonist (GHA). Hormone deficiency such as adrenal insufficiency, hypothyroidism, and hypogonadism was defined when the patients had been received hormone replacement therapy due to symptoms related to hormone deficiency, and low associated hormone levels. For the diagnose of GH deficiency, among the 36 patients who had met the biochemical disease control of acromegaly, 5 (14%) had undergone insulin tolerance test and 6 (17%) had undergone GH-releasing peptides (GHRP)-2 test (18). The number of patients receiving hormone replacement therapy and the total number of replacement therapy for deficient hormones were reviewed using medical records. Hydrocortisone [7 patients (9%)], levothyroxine [11 patients (14%)], and gonadal hormones [3 patients (4%)] were each replaced in patients who had deficient symptoms and corresponding low hormone levels. Only one patient had been diagnosed with GH deficiency and received recombinant GH replacement therapy. Therefore, the IGF-I levels of this one patient were excluded from the present analysis. We were able to include the size of the pituitary tumor before the initial treatment in 64 patients based on imaging data of MRI scans, while 10 patients could not be included due to lack of original data.

The present study adhered to the principles of the Declaration of Helsinki and was approved by the Ethics Committee of Kobe University Hospital (approval #1363).

### Hormone Assay

The serum GH and IGF-I levels were measured using an enzyme-linked immunosorbent assay (ELISA, Tosoh Co. Ltd, Tokyo, Japan) and an immunoradiometric assay (IRMA, Daiichi Radioisotope Laboratories, Tokyo, Japan), respectively. IGF-I SDS was calculated based on age- and sex-matched healthy Japanese individuals, as previously described (19).

### The QoL Questionnaires

In this study, we used the Japanese version of AcroQoL developed from the original Spanish version in collaboration with Dr. S. Webb, a disease-specific QoL assessment (15). The AcroQoL consists of 22 items and two major categories: physical (8 items) and psychological (14 items) scales. The psychological scale is further divided into two subscales; appearance (7 items) and personal relationship (7 items). Each item is answered on a 1–5 Likert scale measuring either the frequency of occurrence or the degree of agreement with the items. The results are quoted as scores in the range of 0–100%, with higher scores indicating better QoL.

The SF-36 was used to measure general health-related QoL, as previously described (20). The SF-36 consists of 36 items evaluating general wellbeing using a 1–5 Likert scale. For the SF-36 Version 2, the results of the items are formulated into two component summaries: the physical component summary (PCS) and the mental component summary (MCS). However, as the factor structure underlying these two-component summaries does not apply to the Japanese population, three-component summaries, including the role/social component summary (RCS), are employed in the Japanese version (21), which was used in the present study. The response for each item was converted to a score within the range of 0–100, with a higher score indicating better QoL.

### Comparison of the Young and Middle-Aged, and Older Patients

We divided all the patients into two age groups based on the World Health Organization definition of older adults (22). The young and middle-aged group included patients younger than 65 years old, and the older group included patients aged 65 years and older. We compared the AcroQoL scores of the groups and investigated the factors associated with QoL impairment.

### The Disease Control Status and Treatment Modalities

We further divided the patients into biochemically controlled or uncontrolled subgroups, depending on the criteria described in *patients and designs*, to determine the effects of disease control status on the AcroQoL score for each age group.

To investigate the association between treatment modalities and the AcroQoL score, we divided the patients into the following three subgroups: surgery only, medical therapy without radiotherapy, and radiotherapy in both age groups.

### Statistical Analysis

All statistical analyses were performed using SPSS ver. 27.0 software (IBM Corporation, Armonk, NY, USA). All continuous variables were analyzed for graphical statistics, and the Shapiro-Wilk normality test was performed to determine the normality of their distribution. The normally or non-normally distributed data for the two groups were compared using the unpaired Student’s *t*-test or the Mann–Whitney *U* test, respectively. A one-way analysis of variance (ANOVA) or Kruskal-Wallis tests with *post-hoc* test was used to compare the intergroup differences of more than three groups in normally or non-normally distributed data, respectively. The χ^2^ test or Fisher’s exact test was used for categorical data. Univariate regression analyses were performed to estimate the variables associated with the impairment of the total and subscale AcroQoL scores. Furthermore, variables with a *P*-value of less than 0.05 in the univariate analyses, were selected as candidates for stepwise multivariate regression analyses. In terms of collinearity between variables, either the total number of hormone replacement or the type of hormone replacement was used as the candidate in the multivariate regression analyses. For the univariate or multivariate regression analyses, the categorical data were converted to dummy variables: absence =0 or presence =1. Similarly, the treatment modalities were coded as dummy variables: surgery only =1, medical therapy without radiotherapy =2, and radiotherapy =3. The results are presented as mean ± standard deviation for normally distributed data and as median [interquartile range] for non-normally distributed data. Statistical significance was set at *P <*0.05.

## Results

### Patient Characteristics

The median age at the time of evaluation was 62.0 [50.7–70.0] years. The male-to-female ratio was approximately 1:1, and the median disease duration was 10.0 [3.0–16.0] years. Since these participants have already been treated, the median random GH and IGF-I SDS were 0.9 [0.3–1.8] ng/mL and 0.1 [-0.8–1.4], respectively. The treatment modalities were as follows: transsphenoidal surgery (TSS) only (n =34, 46%), medical therapy without radiotherapy (n =31, 42%), and radiotherapy (n =9, 12%). Of the 31 patients receiving medical therapy without radiotherapy, 22 were treated with previous TSS, while nine were treated with primary medical therapy. In the radiotherapy subgroup, eight of nine patients were still on medical therapy, while one patient was not receiving drugs for acromegaly. Of all the patients, 36 (48%) achieved biochemical control. The symptoms related to acromegaly were shown in **Table 1**.

**Table 1:** Baseline characteristics of all patients.

N = 74		
age (y)	62.0	[50.7 – 70.0]
gender (male/female)	35/39	
BMI (kg/m^2^)	24.7 ± 4.2	
duration of illness (y)	10.0	[3.0 – 16.0]
age at diagnosis (y)	48.4 ± 12.6	
random GH (ng/mL)	0.9	[0.3 – 1.8]
IGF-I (ng/mL)	128	[100 – 185]
IGF-I SDS	0.1	[-0.8 – 1.4]
size of pituitary tumor (cm)	1.3	[0.9 – 1.8]
**comorbidities and symptoms**		
enlargement of limbs	63 (85%)	
macroglossia	34 (46%)	
headache	22 (29%)	
arthropathy	21 (28%)	
visual impairment	13 (17%)	
hypertension	42 (56%)	
dyslipidemia	32 (43%)	
diabetes mellitus	24 (32%)	
sleep apnea syndrome	20 (27%)	
malignant tumor	12 (16%)	
cardiovascular disease	1 (1%)	
**treatment**		
surgery only	34 (46%)	
medical therapy without radiotherapy	31 (42%)	
medical therapy after surgery	22 (30%)	
primary medical therapy	9 (12%)	
radiotherapy	9 (12%)	
radiotherapy and medical therapy after surgery	8 (10%)	
radiotherapy after surgery	1 (1%)	
drug details		
SRLs alone	19 (25%)	
DAs alone	7 (9%)	
SRLs + DAs	11 (15%)	
SRLs + GHA	1 (1%)	
DAs + GHA	1 (1%)	
replacement therapy		
hydrocortisone	7 (9%)	
levothyroxine	11 (14%)	
recombinant GH	1 (1%)	
gonadal hormone	3 (4%)	
controlled by surgery only	23 (31%)	
controlled by medical therapy without radiotherapy	10 (13%)	
controlled by radiotherapy	3 (4%)	

Normally distributed variables were described as mean ± standard deviation, while non-normally distributed variables were described as median values and interquartile range. Patients with random GH <1.0 ng/mL and IGF-Ⅰ standard deviation score (SDS) <2.0 were defined as controlled.

BMI, body mass index; GH, growth hormone; IGF-Ⅰ, insulin-like growth factor Ⅰ; SRLs, somatostatin receptor ligands; DAs, dopamine agonists; GHA, a growth hormone antagonist.

### Association Between Age and QoL

**Figure 1:**
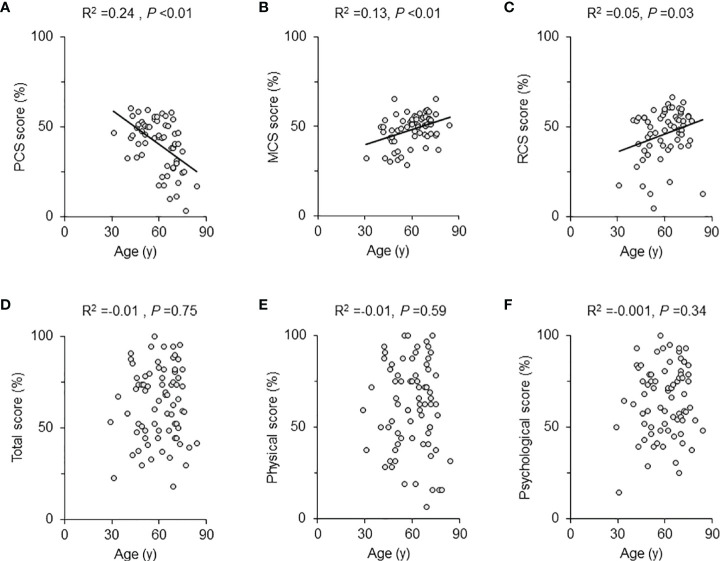
Scatter plot and univariate regression analysis for QoL scores and age. The univariate regression analysis results for the SF-36 scores and age of all patients were shown with scattered plots for each scale: **(A)** physical component summary (PCS) score, **(B)** mental component summary (MCS) score, and **(C)** role/social component summary (RCS) score. The AcroQoL scores of all patients are presented as follows: **(D)** total score, **(E)** physical score, and **(F)** psychological score. The solid lines are approximate straight lines. The adjusted R^2^ and *P*-values are shown in each figure.

In the AcroQoL (n =74), all the scores, including the total, physical, and psychological, were low (63.4 ± 19.8%, 61.1 ± 23.9%, and 64.8 ± 19.1%, respectively) (**Table 2**), as in previous report (23). In the SF-36 (n =61), the PCS score was negatively associated with age (adjusted R^2^ =0.24, *P <*0.01), while both MCS and RCS scores were positively associated with age (adjusted R^2^ = 0.13, *P <*0.01, adjusted R^2^ =0.05, *P* =0.03, respectively) (**Figures 1A–C**). However, no association between age and each AcroQoL score was detected (**Figures 1D–F**).

**Table 2:** QoL scores of 74 patients..

AcroQoL score (%) of 74 patients	
Total	63.4 ± 19.8
Physical	61.1 ± 23.9
Psychological	64.8 ± 19.1
Appearance	57.6 ± 20.5
Personal relationships	72.1 ± 20.9
**SF-36 score (%) of 61 patients**	
Physical component summary	40.2 ± 14.1
Mental component summary	48.2 ± 8.6
Role/social component summary	46.3 ± 13.8

Each score was described as mean ± standard deviation.

### Comparison Between the Young and Middle-Aged, and Older Patients

Next, we divided the patients into two groups, namely the young and middle-aged, and older groups, as shown in the *Materials and Methods* section. As expectedly, both age (at QoL questionnaires) and age at diagnosis were significantly younger in the young and middle-aged group than in the older (*P <*0.01, *P <*0.01, respectively). Their female to male ratio, and serum GH, were not different. Although IGF-I levels were higher in young and middle-aged group, there was no difference in IGF-I SDS. BMI was higher in the young and middle-aged group than in the older (*P* =0.02). Moreover, duration of illness was shorter in the young and middle-aged group than in the older (*P* =0.02). The prevalence of macroglossia, arthropathy, and diabetes mellitus was higher in the older group (*P <*0.01, *P <*0.01, *P <*0.01, respectively). The proportion of treatment modalities did not differ in these groups (**Table 3**).

**Table 3:** Comparison of clinical characteristics between the young and middle-aged, and the older groups.

	young and middle-aged		older		*P*
	N = 42		N = 32		
age (y)	51.0	[46.0 – 59.2]	70.5	[69.0 – 73.0]	**<0.01**
gender (male/female)	22/20		13/19		0.31
BMI (kg/m^2^)	25.7 ± 5.0		23.4 ± 2.5		**0.02**
duration of illness (y)	8.00	[2.7 – 13.2]	12.5	[5.2 – 24.2]	**0.02**
age at diagnosis (y)	42.3 ± 10.9		56.9 ± 9.6		**<0.01**
random GH (ng/mL)	0.6	[0.2 – 1.9]	1.0	[0.6 – 1.8]	0.35
IGF-I (ng/mL)	161	[110 – 193]	110	[81.0 – 143]	**<0.01**
IGF-I SDS	0.3	[-0.8 – 1.5]	-0.03	[-0.9 – 1.2]	0.65
size of pituitary tumor (cm)	1.4	[1.0 – 1.9]	1.2	[0.9 – 1.7]	0.16
**comorbidities and symptoms**					
enlargement of limbs	37 (88%)		26 (81%)		0.46
macroglossia	15 (35%)		19 (59%)		**<0.01**
headache	15 (35%)		7 (21%)		0.26
arthropathy	4 (9%)		17 (53%)		**<0.01**
visual impairment	9 (21%)		4 (12%)		0.32
hypertension	23 (54%)		19 (59%)		0.69
dyslipidemia	15 (35%)		17 (53%)		0.13
diabetes mellitus	8 (19%)		16 (50%)		**<0.01**
sleep apnea syndrome	13 (31%)		7 (21%)		0.38
malignant tumor	4 (9%)		8 (25%)		0.07
cardiovascular disease	1 (2%)		0 (0%)		0.56
**treatment**					
surgery only	22 (52%)		12 (37%)		0.20
medical therapy without radiotherapy	16 (38%)		15 (46%)		0.44
radiotherapy	4 (9%)		5 (15%)		0.33
drug details					
SRLs alone	10 (23%)		9 (28%)		0.67
DAs alone	3 (7%)		4 (12%)		0.35
SRLs + DAs	5 (12%)		6 (18%)		0.31
SRLs + GHA	1 (2%)		0 (0%)		0.56
DAs + GHA	1 (2%)		0 (0%)		0.56
replacement therapy					
hydrocortisone	3 (7%)		4 (12%)		0.35
levothyroxine	5 (12%)		6 (18%)		0.31
recombinant GH	0 (0%)		1 (3%)		0.43
gonadal hormone	3 (7%)		0 (0%)		0.17
controlled by surgery only	15 (35%)		8 (25%)		0.32
controlled by medical therapy without radiotherapy	5 (12%)		5 (15%)		0.44
controlled by radiotherapy	2 (4%)		1 (3%)		0.60

Normally distributed variables were described as mean ± standard deviation, while non-normally distributed variables were described as median values and interquartile range. Patients with random GH <1.0 ng/mL and IGF-Ⅰ standard deviation score (SDS) <2.0 were defined as controlled.

BMI, body mass index; GH, growth hormone; IGF-Ⅰ, insulin-like growth factor Ⅰ; SRLs, somatostatin receptor ligands; DAs, dopamine agonists; GHA, a growth hormone antagonist. In bold: P values less than 0.05.

### The Treatment Status and QoL in Young and Middle-Aged, and Older Patients

**Figure 2:**
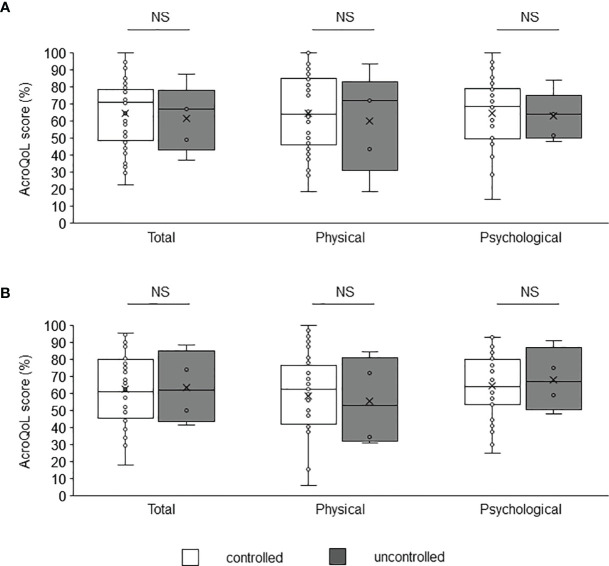
Comparison of AcroQoL scores of the subgroups based on the disease control status. Comparison of AcroQoL scores of the subgroups based on the disease control status in the **(A)** young and middle-aged, and **(B)** older groups. Patients with random GH levels of <1.0 ng/mL and IGF-I SDS of <2.0 are considered controlled, and others are considered uncontrolled. Individual scores have been plotted, and median values and interquartile ranges are shown in the box plot. NS, not significant.

**Figure 3:**
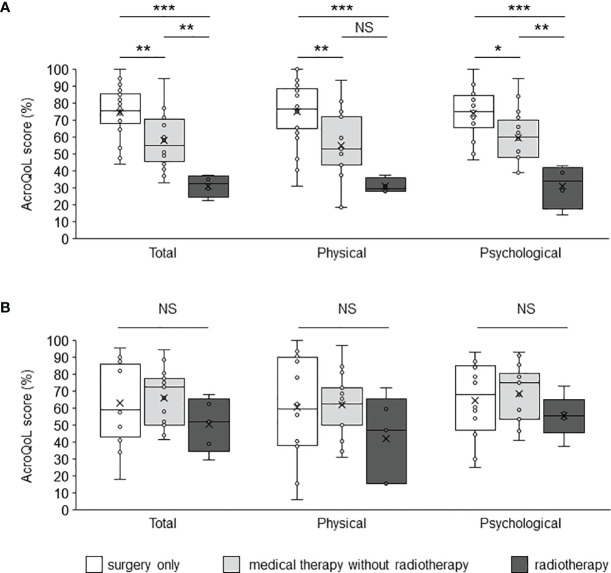
Comparison of the AcroQoL scores of the subgroups based on treatment modalities. Comparison of the AcroQoL scores of the subgroups treated with surgery only, medical therapy without radiotherapy, and radiotherapy in the **(A)** young and middle-aged, and **(B)** older groups. Individual scores have been plotted, and the median values and interquartile ranges are presented in a box plot. NS, not significant, *P < 0.05, **P < 0.01, ***P < 0.001.

A comparison of the AcroQoL scores of the patients with and without biochemical disease control showed no difference in the scores for the young and middle-aged (**Figure 2A**) or older groups (**Figure 2B**). Next, the AcroQoL scores for the following three subgroups were compared based on the treatment modalities: surgery only, medical therapy without radiotherapy, and radiotherapy. In the young and middle-aged group, all the subscale scores of the AcroQoL were higher for patients treated with surgery than for those treated with medical therapy without radiotherapy (**Figure 3A**). The patients who received radiotherapy had the lowest total and psychological QoL scores, as shown previously (15). On the contrary, no difference in the AcroQoL scores was observed among the treatment modality subgroups in the older group (**Figure 3B** **)**.

### Factors Associated With AcroQoL Scores in Young and Middle-Aged, and Older Patients

Stepwise multivariate regression analyses were performed to determine the factors associated with impaired QoL for each age group. In the young and middle-aged group, the treatment modalities (defined in *Material and Methods*), size of pituitary tumor, hydrocortisone replacement therapy, and the total number of hormone replacement therapy were selected as candidate for independent variables by the univariate regression analysis (**Tables S1–S3**). When hydrocortisone replacement therapy, in addition to the treatment modalities and size of pituitary tumor, was included for the multivariate analysis, all the variables were found to be independent factors associated with impaired AcroQoL scores in the young and middle-aged group (**Table 4A**). When the total number of hormone replacement therapy was included in place of hydrocortisone replacement therapy for multivariate analysis, the treatment modalities and the total number of hormone replacement therapy were independently associated with poor AcroQoL scores in total scales (adjusted R^2^ =0.43, *P <*0.01, *P* =0.03, respectively).

**Table 4:** Multivariate regression analysis of determinants of AcroQoL score.

(A) Young and middle-aged						
	Model	adjusted R^2^	independent variables	β	B (95%CI)	*P*
**Total score**	1	0.37	treatment modalities	-0.62	-19.0 (-26.7 – -11.4)	<0.01
	2	0.42	treatment modalities	-0.59	-17.9 (-25.3 – -10.4)	<0.01
			size of pituitary tumor	-0.25	-0.47 (-0.93 – -0.01)	0.04
**Physical score**	1	0.32	treatment modalities	-0.58	-20.9 (-30.3 – -11.5)	<0.01
	2	0.39	treatment modalities	-0.54	-19.3 (-28.4 – -10.3)	<0.01
			size of pituitary tumor	-0.28	-0.64 (-1.2 – -0.07)	0.02
**Psychological score**	1	0.39	treatment modalities	-0.64	-18.8 (-26.0 – -11.6)	<0.01
	2	0.44	treatment modalities	-0.56	-16.4 (-23.7 – -9.1)	<0.01
			hydrocortisone replacement therapy	-0.25	-18.9 (-37.6 – -0.2)	0.04
**(B). Older group**						
	**Model**	**adjusted R^2^**	**independent variables**	**β**	**B (95%CI)**	***P***
**Total score**	1	0.29	arthropathy	-0.56	-23.1 (-36.5 – -9.7)	<0.01
	2	0.41	arthropathy	-0.48	-19.7 (-32.1 – -7.2)	<0.01
			BMI	-0.38	-3.1 (-5.6 – -0.6)	0.01
**Physical score**	1	0.26	arthropathy	-0.54	-27.6 (-44.6 – -10.6)	<0.01
	2	0.37	arthropathy	-0.42	-21.8 (-38.4 – -5.2)	0.01
			hydrocortisone replacement therapy	-0.36	-30.7 (-57.8 – -3.6)	0.02
	3	0.53	arthropathy	-0.30	-15.5 (-30.4 – -0.6)	0.04
			hydrocortisone replacement therapy	-0.46	-39.1 (-63.1 – -15.2)	<0.01
			BMI	-0.43	-4.3 (-7.2 – -1.5)	<0.01
**Psychological score**	1	0.26	arthropathy	-0.54	-27.6 (-44.6 – -10.6)	<0.01
	2	0.35	arthropathy	-0.47	-24.0 (-40.4 – -7.5)	<0.01
			BMI	-0.33	-3.3 (-6.5 – -0.08)	0.04

Stepwise multivariate regression analysis of AcroQoL scores and associated factors (A) in the young and middle-aged group and (B) in the older group. R^2^, coefficient of determination; b, standardized partial regression coefficient; B, unstandardized partial regression coefficient; CI, confidence interval; BMI, body mass index.

In the older group, BMI, complications of dyslipidemia, arthropathy, and hydrocortisone replacement therapy were the independent variables. Interestingly, higher BMI, complications of arthropathy, and hydrocortisone replacement therapy were identified as independent factors contributing to impaired AcroQoL scores in the older group (**Table 4B**).

In recent years, many people over the age of 65 years maintain their physical and mental health and participate in social activities, and some countries including Japan have proposed redefining “older people” as over the age of 75 years (24). Therefore, to clarify the effect of the aging on QoL, all the patients were further divided into the following four groups: younger than 55, 55-64, 65-74, and 75 years and older (**Table S4**). Multivariate regression analysis showed that only the treatment modalities were associated with impaired total AcroQoL scores in both groups <55 and 55-64 years (adjusted R^2^ =0.40, *P <*0.01, adjusted R^2^ =0.35, *P* <0.01, respectively). In the 65-74 years group, higher BMI and complication of arthropathy were associated with poor AcroQoL scores in total scale (adjusted R^2^ =0.47, *P <*0.01, *P* =0.02, respectively). Moreover, only complication of arthropathy was associated in the 75 years and older group (adjusted R^2^ =0.59, *P* =0.02).

## Discussion

This is the first study of the QoL focused on older patients with acromegaly. We demonstrated differences in the factors associated with impaired QoL for different age groups. To the best of our knowledge, most QoL studies focus on young or middle-aged patients with acromegaly, but not on older acromegaly patients. Because of the increasing number of older patients with acromegaly, it is important to adjust the strategy with consideration of QoL.

Age-dependent QoL decline has been observed in healthy populations (12). In this study, age was negatively associated with QoL scores for the physical component of the SF-36; however, a decrease in the QoL for the mental and role/social components was observed in younger patients. These data suggest that acromegaly can have a greater impact on younger patients, especially mentally and socially, rather than physically. On the other hand, AcroQoL showed no association with age, suggesting that age has little effect on the disease-specific QoL for acromegaly. Moreover, the scores of all the patients were impaired.

In this study, we showed that the factors associated with impaired AcroQoL were significantly different for various age groups. For young and middle-aged patients, treatment modalities including current medical therapy and past radiotherapy impaired the QoL score, as shown in previous reports (13, 14). In contrast, treatment modalities did not affect the AcroQoL of older patients.

In the present study, 31 (79%) of 39 patients receiving medical therapy used somatostatin analogs; however, there was no significant difference in the proportion of patients using somatostatin analogs between the younger and older groups. It has been reported that treatment-related burdens such as gastrointestinal side effects, injection site reactions, and the need for periodic drug injections interfere with their daily and work activities, even for the biochemically controlled status of somatostatin analogs (25). The treatment-related burden can lead to QoL impairment for younger patients because of their social status. Moreover, patients who require radiotherapy generally have aggressive tumors, which are often associated with hypopituitarism and may result in impaired QoL.

In contrast, arthropathy and higher BMI were associated with poor QoL for older patients. Motor disability and joint symptoms, mainly due to arthropathy, contribute to reduced AcroQoL scores, even after long-term disease remission (26, 27). Cartilage hypertrophy caused by a chronic excess of GH/IGF-I is partially reversible during the early stages; however, the subsequent deterioration of the joint architecture is irreversible (3, 28). The risk factors for acromegalic arthropathy include female sex, higher BMI, and older age at diagnosis, which are common to primary osteoarthritis (29). In addition, higher GH/IGF-I levels at diagnosis and longer duration of the disease have been demonstrated as disease-specific risk factors for arthropathy (30, 31). In this study, there was no difference in the female ratio between the younger and older groups, and BMI was lower in the older group. It is considered that a longer disease duration in the older group can result in the deterioration of arthropathy. These data suggest the importance of early diagnosis and appropriate treatment of acromegaly to prevent the development of arthropathy.

Interestingly, it has already been reported that a higher BMI is associated with poor QoL in acromegaly (32, 33). A higher BMI is a risk factor for arthropathy and can worsen the symptoms; however, we did not observe an association between the prevalence of arthropathy and BMI. High GH/IGF-I levels in acromegaly can lead to increased muscle mass and extracellular fluid volume, resulting in increased BMI. In contrast, decreased GH/IGF-I levels can promote body mass *via* an increase in fat mass (34). Therefore, the association between chronic GH/IGF-I excess and normalized hormonal levels and an increase in BMI has not been established and remains controversial. The reason for the high BMI contribution to reduced QoL remains unclear. It was speculated that the burden of these comorbidities was more severe in older patients with a higher BMI than in younger patients.

Excess GH/IGF-I in acromegaly causes insulin resistance and glucose intolerance, increasing the risk of frailty or sarcopenia, especially in older patients (35). The presence of sarcopenia is associated with decrease in health-related QoL (36). Moreover, a decrease in IGF-I after treatment for acromegaly has been shown to increase fat accumulation and decrease lean body mass (37). Multiple factors, including changes in body composition and sarcopenia, may affect health-related QoL in older acromegaly patients under treatment. Further studies focused on the effect of these metabolic factors on QoL in older patients are needed.

In the present study, the number of hormone replacement therapy was associated with poor AcroQoL scores in young and middle-aged group. It was reported that patients with multiple pituitary hormone deficiencies had worse QoL than patients with isolated GH deficiency (38). However, there was no association between the number of hormone replacement therapy and the AcroQoL scores in older patients in this study. Further investigation is needed to clarify whether impairment of QoL due to multiple pituitary hormone deficiencies varies with age.

In contrast, the AcroQoL scores were lower for patients who received hydrocortisone replacement therapy in both age groups in this study. It is well-known that patients with adrenal insufficiency, compared with the general population, have impaired health-related QoL due to exhaustion, social disability, and anxiety irrespective of age, even with hydrocortisone replacement therapy (39). Therefore, it is not surprising that hydrocortisone replacement therapy was associated with lower AcroQoL scores in both age groups in the present study.

It was interesting that the size of pituitary tumor before the initial treatment was associated with poor AcroQoL scores in young and middle-aged patients, while no association was found in older group in this study. It was reported that macroadenoma impaired health-related QoL in the patients with non-functional pituitary tumor (40). Although pathological findings were not reviewed in the present study, it was possible that several patients in the young and middle-aged group with macroadenoma had sparsely granulated adenoma, which was resistant to treatment, leading to decreased QoL.

The limitation of the present study is that it was a retrospective cross-sectional study with a relatively small sample. Therefore, improvements in QoL associated with early diagnosis and appropriate treatment of arthropathy remain to be elucidated, although arthropathy is generally irreversible. Moreover, in this study, we only investigated the acromegaly patients without a control study using healthy subjects. To conclude the effect of aging on QoL with acromegaly patients under treatment, we needed further study including control subjects of young, middle-aged, and older people.

In conclusion, we have shown that the factors differently affected QoL across the age groups. Arthropathy, higher BMI, and hydrocortisone replacement therapy are associated with impaired disease-specific QoL of older patients with acromegaly. These data suggest that early diagnosis and appropriate treatment are important to prevent impaired QoL of older patients.

## Data Availability Statement

The raw data supporting the conclusions of this article will be made available by the authors, without undue reservation.

## Ethics Statement

The studies involving human participants were reviewed and approved by the Ethics Committee of Kobe University Hospital (approval #1363). Written informed consent for participation was not required for this study in accordance with the national legislation and the institutional requirements.

## Author Contributions

NY, SU, HF, and YT conceived and designed the study. NY, SU, KY, MY, and HF contributed to collect the data. NY, SU, MY, HF, and GI contributed to analyze data. NY, SU, HF, MY, MS, HS, YF, KK, GI, YT, and WO interpreted data and contributed to discussion. NY, SU, HF, and YT drafted the manuscript.

## Funding

This work was supported by Grant-in-Aid for Scientific Research from the Japanese Ministry of Education, Culture, Sports, Science and Technology 19K09003.

## Conflict of Interest

The authors declare that the research was conducted in the absence of any commercial or financial relationships that could be construed as a potential conflict of interest.

## Publisher’s Note

All claims expressed in this article are solely those of the authors and do not necessarily represent those of their affiliated organizations, or those of the publisher, the editors and the reviewers. Any product that may be evaluated in this article, or claim that may be made by its manufacturer, is not guaranteed or endorsed by the publisher.
