# Supplementary material for: The Effect of Aging on Quality of Life in Acromegaly Patients Under Treatment
Source: Front Endocrinol (Lausanne). 2022 Feb 3;13:819330. doi: 10.3389/fendo.2022.819330 (PMC8850938; doi:10.3389/fendo.2022.819330)
Supplement: Supplementary file 1 [file DataSheet_1.docx]

**TableS-1. Univariate regression analysis of determinants of AcroQoL total score.**

|  | **Young and middle-aged** | | **Older** | |
| --- | --- | --- | --- | --- |
| **independent variables** | **adjusted R^2^** | ***P*** | **adjusted R^2^** | ***P*** |
| age | 0.03 | 0.14 | < 0.01 | 0.33 |
| gender | < 0.01 | 0.29 | -0.03 | 0.78 |
| BMI | 0.04 | 0.19 | **0.21** | **< 0.01** |
| duration of illness | -0.01 | 0.59 | -0.03 | 0.78 |
| random GH | < 0.01 | 0.93 | 0.04 | 0.13 |
| IGF-Ⅰ SDS | 0.02 | 0.80 | -0.03 | 0.94 |
| size of pituitary tumor | **0.09** | **0.03** | -0.04 | 0.92 |
| enlargement of limbs | < 0.01 | 0.34 | < 0.01 | 0.35 |
| macroglossia | -0.02 | 0.60 | < 0.01 | 0.76 |
| headache | -0.02 | 0.66 | 0.01 | 0.24 |
| arthropathy | -0.02 | 0.83 | **0.29** | **< 0.01** |
| visual impairment | 0.03 | 0.13 | 0.01 | 0.23 |
| hypertension | 0.04 | 0.10 | -0.02 | 0.52 |
| dyslipidemia | 0.01 | 0.24 | 0.08 | 0.06 |
| diabetes mellitus | -0.01 | 0.44 | -0.03 | 0.96 |
| sleep apnea syndrome | 0.05 | 0.07 | < 0.01 | 0.39 |
| malignant tumor | -0.02 | 0.63 | -0.01 | 0.48 |
| treatment modalities | **0.42** | **< 0.01** | -0.01 | 0.41 |
| total number of replacement therapy | **0.16** | **< 0.01** | 0.04 | 0.14 |
| hydrocortisone replacement therapy | **0.14** | **< 0.01** | 0.08 | 0.05 |
| levothyroxine replacement therapy | 0.03 | 0.12 | -0.02 | 0.52 |

Univariate regression analyses were performed to estimate the variables associated with the impairment of the total AcroQoL score. Variables with a *P*-value of <0.05 were used as candidates for multivariate regression analysis. R^2^; coefficient of determination.

**Table S-2. Univariate regression analysis of determinants of AcroQoL physical score.**

|  | **Young and middle-aged** | | **Older** | |
| --- | --- | --- | --- | --- |
| **independent variables** | **adjusted R^2^** | ***P*** | **adjusted R^2^** | ***P*** |
| age | < 0.01 | 0.26 | 0.08 | 0.05 |
| gender | < 0.01 | 0.34 | -0.03 | 0.83 |
| BMI | -0.01 | 0.45 | **0.15** | **0.02** |
| duration of illness | -0.02 | 0.85 | -0.01 | 0.47 |
| random GH | -0.02 | 0.90 | 0.04 | 0.14 |
| IGF-Ⅰ SDS | -0.02 | 0.64 | -0.03 | 0.91 |
| size of pituitary tumor | **0.11** | **0.01** | -0.04 | 0.92 |
| enlargement of limbs | < 0.01 | 0.36 | -0.01 | 0.46 |
| macroglossia | < 0.01 | 0.30 | -0.04 | 0.91 |
| headache | -0.02 | 0.94 | < 0.01 | 0.36 |
| arthropathy | -0.02 | 0.62 | **0.26** | **< 0.01** |
| visual impairment | < 0.01 | 0.28 | -0.02 | 0.52 |
| hypertension | < 0.01 | 0.25 | -0.01 | 0.43 |
| dyslipidemia | 0.01 | 0.23 | **0.13** | **0.02** |
| diabetes mellitus | < 0.01 | 0.39 | -0.03 | 0.96 |
| sleep apnea syndrome | 0.04 | 0.09 | < 0.01 | 0.31 |
| malignant tumor | < 0.01 | 0.32 | -0.03 | 0.82 |
| treatment modalities | **0.37** | **< 0.01** | < 0.01 | 0.28 |
| total number of replacement therapy | **0.10** | **0.02** | 0.08 | 0.06 |
| hydrocortisone replacement therapy | **0.07** | **0.04** | **0.15** | **0.01** |
| levothyroxine replacement therapy | < 0.01 | 0.30 | -0.01 | 0.50 |

Univariate regression analyses were performed to estimate the variables associated with the impairment of the physical AcroQoL score. Variables with a *P*-value of <0.05 were used as candidates for multivariate regression analysis. R^2^; coefficient of determination.

**Table S-3. Univariate regression analysis of determinants of AcroQoL psychological score.**

|  | **Young and middle-aged** | | **Older** | |
| --- | --- | --- | --- | --- |
| **independent variables** | **adjusted R^2^** | ***P*** | **adjusted R^2^** | ***P*** |
| age | 0.04 | 0.10 | -0.03 | 0.90 |
| gender | < 0.01 | 0.30 | -0.02 | 0.56 |
| BMI | 0.04 | 0.12 | **0.21** | **< 0.01** |
| duration of illness | < 0.01 | 0.32 | -0.03 | 0.94 |
| random GH | -0.02 | 0.95 | 0.03 | 0.16 |
| IGF-Ⅰ SDS | -0.02 | 0.94 | -0.03 | 0.79 |
| size of pituitary tumor | 0.06 | 0.06 | -0.04 | 0.79 |
| enlargement of limbs | < 0.01 | 0.37 | < 0.01 | 0.30 |
| macroglossia | -0.02 | 0.88 | -0.03 | 0.64 |
| headache | -0.01 | 0.51 | 0.03 | 0.18 |
| arthropathy | -0.02 | 0.99 | **0.21** | **< 0.01** |
| visual impairment | 0.04 | 0.09 | 0.05 | 0.11 |
| hypertension | 0.05 | 0.06 | -0.02 | 0.69 |
| dyslipidemia | < 0.01 | 0.28 | 0.02 | 0.18 |
| diabetes mellitus | -0.01 | 0.52 | -0.03 | 0.95 |
| sleep apnea syndrome | 0.04 | 0.09 | -0.02 | 0.52 |
| malignant tumor | -0.02 | 0.93 | 0.03 | 0.16 |
| treatment modalities | **0.39** | **< 0.01** | -0.02 | 0.55 |
| total number of replacement therapy | **0.17** | **< 0.01** | < 0.01 | 0.28 |
| hydrocortisone replacement therapy | **0.16** | **< 0.01** | 0.03 | 0.16 |
| levothyroxine replacement therapy | 0.05 | 0.08 | -0.02 | 0.57 |

Univariate regression analyses were performed to estimate the variables associated with the impairment of the psychological AcroQoL score. Variables with a *P*-value of <0.05 were used as candidates for multivariate regression analysis. R^2^; coefficient of determination.

**Table S-4. Comparison of clinical characteristics between the age groups.**

|  | < 55 y | 55-64 y | 65-74 y | 75 ≤ y |
| --- | --- | --- | --- | --- |
|  | N = 26 | N = 16 | N = 25 | N = 7 |
| age (y) | 47.0 [42.7 – 51.0]^†#^ | 60.0 [57.5 – 62.7]^*#^ | 70.0 [68.5 – 71.5]^*†^ | 76.0 [75.0 – 79.0]^*†^ |
| gender (male/female) | 16 / 10 | 6 / 10 | 9 / 16 | 4 / 3 |
| BMI (kg/m^2^) | 25.8 ± 5.3 | 25.5 ± 4.5 | 23.5 ± 2.8 | 22.9 ± 0.6 |
| duration of illness (y) | 7.00 [1.75 – 14.2] | 8.50 [3.00 – 11.7] | 10.0 [4.00 – 19.0] | 21.0 [13.0 –29.0]^*†#^ |
| age at diagnosis (y) | 39.5 [29.7 – 46.2] ^†#^ | 51.5 [47.5 – 54.0]^*^ | 58.5 [51.2 – 64.7]^*^ | 59.0 [49.0 – 64.2]^*^ |
| random GH (ng/mL) | 0.63 [0.19 – 2.03] | 1.20 [0.35 – 2.50] | 0.95 [0.63 – 2.05] | 1.16 [0.29 – 1.81] |
| IGF-Ⅰ (ng/mL) | 161 [115 – 191]^#^ | 167 [104 – 203]^#^ | 110 [89.0 – 153]^*†^ | 99.0 [49.5 – 132]^*†^ |
| IGF-Ⅰ SDS | 0.20 [-0.89 – 1.10] | 1.11 [-0.82 – 1.81] | -0.03 [-0.68 – 1.19] | -0.41 [-2.14 – 0.89] |
| size of pituitary tumor (cm) | 1.59 [1.10 – 2.48] | 1.18 [0.90 – 1.75] | 1.16 [0.80 – 1.69] | 1.31 [1.13 – 1.70] |
| **comorbidities and symptoms** |  |  |  |  |
| enlargement of limbs | 22 (84%) | 1 (6%) | 22 (88%) | 4 (57%) |
| macroglossia | 9 (34%)^#^ | 6 (37%)^#^ | 16 (64%)^†*^ | 3 (43%) |
| headache | 12 (46%) | 3 (18%) | 5 (20%) | 2 (28%) |
| arthropathy | 3 (11%)^#^ | 1 (6%)^#^ | 13 (52%)^*†^ | 4 (57%)^*†^ |
| visual impairment | 7 (27%) | 2 (12%) | 3 (12%) | 1 (14%) |
| hypertension | 9 (34%)^†#^ | 14 (87%)^*^ | 16 (64%)^*^ | 3 (43%)^†^ |
| dyslipidemia | 6 (23%)^†#^ | 9 (56%)^*^ | 13 (52%)^*^ | 4 (57%) |
| diabetes mellitus | 3 (11%)^#^ | 5 (31%) | 12 (48%)^*^ | 4 (57%)^*^ |
| sleep apnea syndrome | 6 (23%) | 7 (43%) | 6 (24%) | 1 (14%) |
| malignant tumor | 1 (3%)^#^ | 3 (18%) | 8 (32%)^*^ | 0 (0%) |
| cardiovascular disease | 0 (0%) | 1 (6%) | 0 (0%) | 0 (0%) |
| **treatment** |  |  |  |  |
| surgery only | 13 (50%) | 9 (56%) | 9 (36%) | 3 (43%) |
| medical therapy  without radiotherapy | 9 (34%) | 7 (43%) | 13 (52%) | 2 (28%) |
| radiotherapy | 4 (15%) | 0 (0%) | 3 (12%) | 2 (28%) |
| replacement therapy |  |  |  |  |
| hydrocortisone | 3 (11%) | 0 (0%) | 1 (4%) | 3 (43%)^†#^ |
| levothyroxine | 5 (19%) | 0 (0%) | 3 (12%) | 3 (43%)^†^ |
| recombinant GH | 0 (0%) | 0 (0%) | 0 (0%) | 1 (14%) |
| gonadal hormone | 3 (11%) | 0 (0%) | 0 (0%) | 0 (0%) |

Normally distributed variables were described as mean ± standard deviation, while non-normally distributed variables were described as median values and interquartile range.

^*^*P*-value of <0.05 when compared to the group < 55 y. ^†^*P*-value of <0.05 when compared to the group 55-64 y. ^#^ *P*-value of <0.05 when compared to the group 65-74 y.

BMI; body mass index, GH; growth hormone, IGF-Ⅰ; insulin-like growth factor Ⅰ.
